# It’s a match!? Appropriate item selection in the Concealed Information Test

**DOI:** 10.1186/s41235-019-0161-8

**Published:** 2019-04-03

**Authors:** Linda Marjoleine Geven, Gershon Ben-Shakhar, Merel Kindt, Bruno Verschuere

**Affiliations:** 1 Faculty of Social and Behavioural Sciences, Department of Clinical Psychology, University of Amsterdam, Amsterdam, The Netherlands; 2 Department of Psychology, Hebrew University of Jerusalem, Jerusalem, Israel

**Keywords:** Memory detection, Deception, External validity, Leakage, Diagnosticity

## Abstract

**Background:**

While the Concealed Information Test (CIT) can determine whether examinees recognize critical details, it does not clarify the origin of the memory. Hence, when unknowledgeable suspects are contaminated with crime information through media channels or investigative interviews, the validity of the CIT can be compromised (i.e. false-positive outcomes). Yet, when the information was disclosed solely at the category level (e.g. the perpetrator escaped in a car), presenting specific items at the exemplar level (e.g. Citroën, Opel, or Volkswagen) might preclude this problem. However, diminished recollection for exemplar-level details could attenuate the CIT effect for knowledgeable suspects, thereby leading to false negatives. The appropriate item level for memory detection to reach an optimal balance between sensitivity and specificity remains elusive. As encoding, retention, and retrieval of information may influence memory performance and thereby memory detection, the current study investigated the validity of the CIT on both categorical and exemplar levels.

**Results:**

Participants planned a mock robbery (*n* = 165), with information encoded at the category (e.g. car) or exemplar (e.g. Citroën) level. They were tested immediately or after a one-week-delay, with a response time-based CIT consisting of questions at the categorical or exemplar level. An interaction was found between encoding and testing, such that CIT validity based on reaction time was higher for “matching” (e.g. exemplar-exemplar) than for “mismatching” (e.g. exemplar-categorical) items, while immediate versus one week delayed testing did not affect the outcome.

**Conclusion:**

Critically, this indicates that what constitutes a good CIT item depends on the way the information was encoded. This provides a challenge for CIT examiners when selecting appropriate items.

**Electronic supplementary material:**

The online version of this article (10.1186/s41235-019-0161-8) contains supplementary material, which is available to authorized users.

## Significance

The purpose of memory detection is to verify whether suspects are aware of critical information related to a crime by measuring psychophysiological or behavioral responses. The method requires that the examiner determines a number of established facts from the investigation to create a multiple-choice like test with several questions, for instance probing whether the murder weapon was a bomb, a firearm, or a knife. However, when factually innocent suspects are aware of the correct answers through contamination in the interrogation or by the news media, they become at risk of being incorrectly classified as knowledgeable. The current study investigated whether this important issue could be circumvented by asking for more specific details, such as the specific type of knife that was used, and how this influenced the rate at which suspects can be correctly classified. Results indicated that what constitutes a good question for memory detection purposes strongly depends on the way the perpetrator remembered the detail. This provides a challenge for examiners when selecting appropriate items on a case-to-case basis.

## Introduction

David Lykken introduced the Guilty Knowledge Test – nowadays known as the Concealed Information Test (CIT; Verschuere, Ben-Shakhar, & Meijer, 2011) – in 1959. The purpose of this alternative to traditional polygraph testing is to verify whether suspects show physiological or behavioral responses signaling recognition of critical knowledge about the crime under investigation. By demonstrating (lack of) awareness of the critical information, the CIT is expected to differentiate between knowledgeable and unknowledgeable suspects. Imagine the following case in which a robber enters a bank in his hometown, points a knife at the teller, and steals a precious jewel from a safety deposit box, while his companion waits outside in the car. The examiner creates a CIT in which, similar to a multiple-choice test with several answering options, critical information is probed while measuring the suspects’ response to all presented stimuli. In this specific case, the examiner might draft a question regarding the getaway vehicle, such as “How did the perpetrator flee from the scene? a) by train, b) by bike, c) by taxi, d) by car, or e) by bus.” A suspect who committed this armed robbery, would recognize the correct option and show enhanced responses to the word “car” compared to the plausible alternatives. On the contrary, an innocent suspect with no means of knowing which alternative is correct is not expected to show any distinct responses to the correct, critical information.

Whereas in the past we had to wait for newspapers to receive the latest novelties, since the dawn of mass media, news spreads easier and faster than ever. When innocents are “contaminated” with crime knowledge, they become at risk of recognizing, and hence responding to, these crime details in the CIT. Indeed, several studies have shown that knowledge in itself – irrespective of its origin – is sufficient to elicit differential responses in the CIT (see a review by Bradley, Barefoot, & Arsenault, 2011). Bradley and Rettinger (1992) compared three groups of participants on their recognition of critical information from a mock crime in which the perpetrator “murdered and robbed” a medical mannequin. As expected, participants who committed the mock crime could be clearly distinguished from innocent participants who had been waiting in the laboratory. However, a third group of informed innocent participants who had merely been notified of details regarding the murder also showed a distinct response upon the presentation of the critical detail. While the CIT is an established method to verify whether an individual possesses critical knowledge, it does not pinpoint the source of this information.

In an attempt to solve this problem of information leakage, the Guilty Action Test (GAT; Bradley & Warfield, 1984) was developed. While retaining the main elements of the CIT, rather than probing for passive knowledge about the crime (e.g. Which vehicle was used to flee from the crime scene?), questions in the GAT are related to actions of the suspect (e.g. How did *you* flee from the scene?). Contrary to the CIT, informed innocents recognizing the correct answer could now remain truthful while denying knowledge of the correct option. Bradley, MacLaren, and Carle (1996) found that the GAT revealed a lower false-positive rate when testing informed innocents than the CIT. However, a more recent direct comparison of the two methods to detect concealed information revealed that similar to the CIT, the GAT could not accurately distinguish between participants who committed the mock crime and innocent participants who were informed about its details (Gamer, 2010).

In Japan, where the CIT is applied in criminal investigations on a large scale (Osugi, 2018), the leakage problem is often tackled by formulating questions at the exemplar level. The crux is to ask for more specific information that is less likely to be leaked to the general public, such as the brand of the getaway vehicle. For instance, if the innocent knows that the perpetrators got away by car, it might not be known whether it was a Citroën, Opel, or Volkswagen. At the same time, asking such specific questions raises new challenges for the CIT regarding the balance between false-positive and false-negative outcomes.

A first challenge concerns (lack of) memory for exemplar details. Examiners have to estimate what the perpetrator experienced during the robbery and choose specific details such that there is great likelihood that a guilty suspect would have noticed them, stored them in memory, and remembers it at the time of the CIT. Specifically, when the interval between crime and memory detection test is large, perpetrators might forget specific details of the crime (Nahari & Ben-Shakhar, 2011). Research findings confirm that a time delay can diminish the detection efficiency of the CIT (Carmel, Dayan, Naveh, Raveh, & Ben-Shakhar, 2003; Gamer, Kosiol, & Vossel, 2010; Nahari & Ben-Shakhar, 2011; Peth, Vossel, & Gamer, 2012), particularly for peripheral details of the crime.

A second challenge concerns item distinguishability, referring to the fact that items at an exemplar level may be less discernable from the alternative fillers than items at a broader category level (Osugi, 2018). Probing exemplar-level critical details in a multiple-choice format requires homogeneously matched control alternatives in the CIT. Since items at the exemplar level are more similar than a set of words at the category level, because they share the same category, this may diminish the capacity of the critical detail to pop-out among the other alternatives (Donchin, 1981; Sokolov, 1963).

Indeed, when the critical detail closely resembled the alternatives it was found that CIT validity decreased significantly (Ben-Shakhar & Gati, 1987). In a recent experiment in which the CIT was used to assess eyewitnesses’ face memory, the CIT effect was nearly eliminated (Sauerland, Wolfs, Crans, & Verschuere, 2017). As the perpetrator was presented within a series of matched foil faces that very well resembled the culprit, the cooperative eyewitnesses did not show the automatic, differential response pattern to the critical face. Hence, stimulus distinctiveness seems to be an important factor for successful memory detection.

Likewise, validity of memory detection is lowered when the items in the CIT slightly differ from the encoded information in modality, category or semantics. A linear relationship was observed between physiological responsivity and the degree of match with the original stimulus, with an identical representation of the critical CIT item and the examinees’ memory revealing the highest detection efficiency (e.g. apple – apple; Ben-Shakhar & Gati, 1987; Ben-Shakhar, Gati, & Salamon, 1995). Still, participants did show a CIT effect upon the presentation of synonyms, pictures of verbally encoded stimuli, or the critical items’ subordinate (e.g. apple – fruit; Ben-Shakhar, Frost, Gati, & Kresh, 1996). Thus, while asking specific questions may help to reduce the risk of false positives associated with the leakage of crime information, it may increase the risk of false negatives due to reduced memory and limited distinguishability between the critical and control items, which may lead to decreased responsivity to the critical item.

In the present study, we further expand existing research on this issue that has both theoretical and practical implications, by investigating what would be the optimal question format for the CIT. While Ben-Shakhar et al. (1996) examined generalization from exemplar level to category level, the present study uses a full cross-over design. Besides testing the optimal level of abstractness for CIT questioning, this allows to investigate whether the CIT detection efficiency depends on the level of abstractness during encoding.

Participant couples were involved in the planning phase of a mock robbery of a bank, with the critical items encoded either at the category (e.g. you will flee from the crime scene in a car) or exemplar (e.g. you will flee from the crime scene in a Citroën) level. One participant of the pair was asked to immediately participate in a deception detection test, while the other participant completed the test after a one-week-delay. In the CIT, half of the items were presented at the congruent abstractness level (either categorical or exemplar), whereas the other half were replaced by the corresponding test stimulus at the incongruent abstractness level, leading to a crossed design that investigates the optimal item selection for memory detection while maintaining both sensitivity and specificity.

## Method

The study was approved by the ethical committee of the Department of Psychology of the University of Amsterdam (2017-CP-7836). The tasks scripts and data are available on https://osf.io/crw7f/, where the pre-registration for the hypotheses, methods, and analyses can also be found.

### Participants

A total of 165 participants (63.6% female) were recruited for this study through a university portal or through advertisements on social media. Their average age was 24.23 years (SD_age_ = 8.44, range 18–61 years). Participants received course credits or a monetary equivalent as compensation. All participants provided consent before taking part in the study.

Seven participants did not complete the full study and were therefore excluded from data analyses. Twenty-two participants were excluded due to low target accuracy on the CIT (i.e. an error rate of 50% or more on target items, see Kleinberg & Verschuere, 2015). This criterion ensured that only those participants who understood the instructions and took the task seriously were included in the data analysis. All participants who completed the CIT had > 50% trials remaining after excluding errors and outliers (see the “Results” section below).

The final sample for analysis consisted therefore of 136 participants (64.7% female, *M*_age_ = 24.70, *SD*_age_ = 9.01). Seventy-one participants (64.8% female, *M*_age_ = 25.48, *SD*_age_ = 9.76) were randomly assigned to the immediate CIT condition and 65 participants (64.6% female, *M*_age_ = 23.85, *SD*_age_ = 8.11) were randomly assigned to the delayed CIT condition, completing the CIT after a one-week interval. There were no significant differences between the immediate and the delayed condition in age, *t* (134) = 1.06, *p* = 0.293, *d*_*between*_ = 0.18 or gender, *X*^*2*^ (1) = 0.00, *p* = 0.983, φ_c_ = 0.00.

### Material

#### Crime scenario

Participants were told that they were going to plan a mock robbery and would work together as partners in crime. The crime scenario consisted of a coherent story based on eight critical details, of which four presented in their categorical form and four in their exemplar form: Participants encoded that they had met each other in the *sports club* (exemplar: *volleyball club*) and planned to rob a *bank* (exemplar: *SNS bank*) in their residence of *South-Holland* (exemplar: *Delft*) in *May* (exemplar: *May 26th*). Because they might not be able to flee the scene without a fight, they would bring a *knife* (exemplar: *butterfly knife*). The partners in crime plan to steal expensive *jewelry* (exemplar: *ring*) and hide it at *home* (exemplar: *attic*). Lastly, they planned to flee the crime scene *by car* (exemplar: *Citroën*; please see Additional file 1 for all possible item combinations).

#### RT-CIT

During the CIT all participants were explicitly instructed to conceal their knowledge of the planned robbery. Participants were required to deny knowledge for trials containing critical details from the plan (i.e. respond “no,” hence lying) while telling the truth to irrelevant items (i.e. respond “no”).

**Fig. 1:**
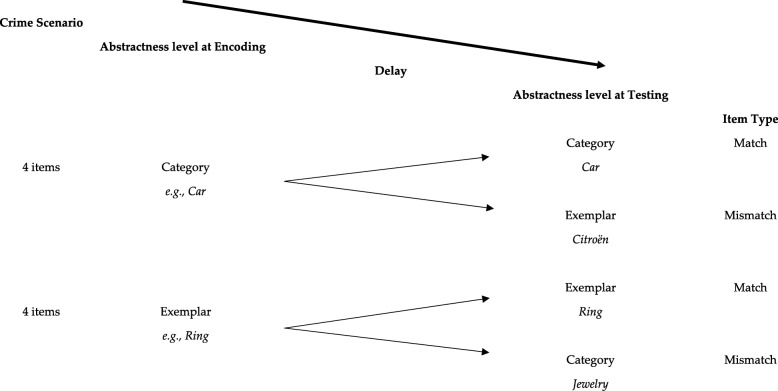
Item distribution in the CIT

All eight critical items were presented at the categorical or exemplar level: two stimuli were encoded at the category level and were also presented at the category level (e.g. encoded as *car*, tested as *car*, congruent with encoding); two stimuli were encoded at the exemplar level and were also presented at the exemplar level (e.g. encoded as *Citroën*, tested as *Citroën*, congruent with encoding). In two other instances, the stimuli encoded at the category level were replaced by the corresponding test stimulus in its exemplar form (e.g. encoded as *car*, tested as *Citroën*; incongruent with encoding, as no exemplar-level information was made available at encoding) and two of the stimuli encoded on the exemplar level were replaced by the corresponding test stimulus in its category form (e.g. encoded as *Citroën*, tested as *car*; incongruent with encoding, as only exemplar-level information was made available at encoding; see also Fig. 1). Lastly, target items (e.g. *train*) were added to ensure that examinees pay attention to all items. These items have to be answered “yes” and were learned just before commencing the CIT. Targets were always presented and tested at the same abstractness level as the critical items in the RT-CIT.

#### Follow-up questionnaire

Motivational states were reported in a questionnaire involving five questions that participants had to rate on a 5-point Likert scale (ranging from 1 = not at all to 5 = very much so). This questionnaire measured how well participants were able to focus on the screen during the CIT, how involved they were in the study, how well their memory was for the items of the planned robbery and the learned target items, as well as how much they tried to avoid detection and appear innocent in the CIT.

#### Recall and recognition

To examine potential differences in memory performance between the immediate and delayed CIT condition, memory for the critical items of the planned robbery were assessed with a free recall followed by a recognition test after the full procedure. Participants first had to freely recall the eight details from the encoding phase, in which they had planned the robbery. In the subsequent recognition test, participants had to pick the correct details from a list of all eight critical items intermixed with irrelevant options (i.e. four irrelevant options per each critical detail, leading to 40 items).

For recall, the number of correctly recalled details were counted, leading to a total score between, this represents the possible range 0–8. For recognition, items were scored as either correct (1) or incorrect (0) and summed, leading to a total score between 0–8.

### Procedure

Participants were invited to come to the laboratory in pairs and work as partners in crime while planning a robbery. When only one participant showed up (*n* = 27), the experimenter took the place of the partner in the planning phase of the mock crime. The experimenter explained that it was important to remember the details from the crime as well as possible and to visualize actually committing the robbery. In order to prevent possible detection, participants were asked to align their stories as much as possible and study the details extensively.

The experimenter first read the plan for the robbery out loud, with its eight critical details presented in either categorical or exemplar level. Participants had to encode all items by writing down the words, reading it out loud and probing each other for the information. During this encoding phase, the experimenter stayed in the room to assure that participants would not accidentally fill in an exemplar-level detail when aligning their story (e.g. inventing a specific date if they had to encode the month *May*). During the encoding phase, the experimenter asked the pair additional questions to stimulate richer encoding of the crime items and to contextualize the critical details (e.g. discuss how long you have known your partner in crime, and who will be driving the getaway vehicle). Then, the experimenter asked participants one by one to repeat the eight critical details from the robbery, until all items were recalled correctly. Lastly, participants filled in the missing details of the story on paper, followed by a free recall of the items. After this 10-min encoding phase, one participant of the pair was randomly assigned to the immediate testing condition, whereas the other participant was assigned to complete the CIT after a one-week delay (± 1 day). Participants were explicitly instructed not to discuss details of the experiment with each other in the one-week period between the encoding phase and the second session.

In the second phase of the experiment, participants were asked to sit behind the computer for the CIT (programmed in Inquisit 4.0). The experimenter explained that the police had received an anonymous tip about an upcoming robbery and that the participant was taken to the police station to undergo a lie detection test. The participant was instructed to try to convince the police of his innocence and beat the lie detector test by hiding all information about the crime. Upon successful concealment, the participant would receive an additional 0.5 course credit compensation.^1^

Then, participants were asked to encode eight target items to which they should respond affirmatively in the CIT, while denying all other information (i.e. both the critical details and irrelevant options). The eight targets were initially presented on the screen for 2 min and participants were asked to recall all items. Then, they saw the targets for an additional 1 min before recalling them again and continuing to the practice phases of the CIT.

For each of the eight critical details encoded in the crime scenario, the CIT included the correct answer, a target item and four incorrect answers serving as irrelevant options (ratio 1:1:4). For instance, if May was the critical stimulus, the target was July and the irrelevant stimuli were June, August, September, and October (categorical Item Type); if May 26 was the critical stimulus, the target was May 30 and the irrelevant stimuli were May 8, May 12, May 17, and May 22 (exemplar Item Type). All eight critical items, eight target items, and 32 irrelevant items were displayed exactly 14 times, leading to a total of 672 trials in the test. These trials were divided over two blocks, each containing 336 trials, with a self-paced break in between. The sequence of the stimuli within the block was completely randomized, following a multiple-probe-protocol (see also Verschuere, Kleinberg, & Theocharidou, 2015).

**Fig. 2:**
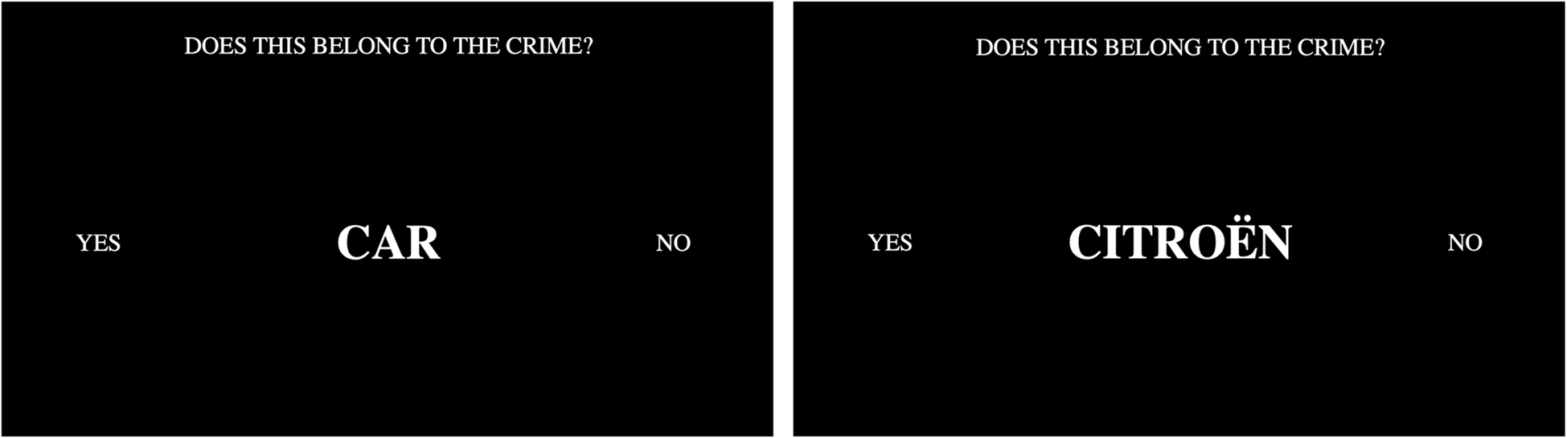
Example CIT trials

During the test, participants had to respond to the question “*Does this belong to the crime?*” by pressing either the left button (A-key) for YES, or the right button (L-key) for NO on their keyboard (see Fig. 2). The question and the response keys remained on the screen during the entire test as a reminder. Participants were instructed to respond with YES only to the target items and NO to all other stimuli (i.e. both the correct details of the planned robbery and the irrelevant options). Each trial consisted of one answer (e.g. June) being displayed as a word in the middle of the screen for exactly 1500 ms. If the participant did not respond within the maximum response deadline of 800 ms, the message TOO SLOW appeared in red above the stimulus for 200 ms. If the participant’s response was incorrect, that is responding with NO for target items or with YES to critical or irrelevant items, the word WRONG appeared in red below the stimulus for 200 ms. Response latency was measured from the onset of the stimulus on the screen until one of the response keys was pressed. After key-press or after the 1500 ms presentation time, the next stimulus appeared on the screen with an inter-stimulus interval (ISI) of either 250, 500, or 750 ms to prevent response preparation.

In order to ensure proper understanding of the task and instructions, each participant had to pass through a stepwise practice procedure that allowed participants to become used to the speed and the requirements of the CIT. Each of the three practice phases of the memory detection test consisted of 24 trials displaying a random subset of critical, irrelevant, and target items. In the first practice phase, participants could pace the speed of the trial sequence themselves, so that a new stimulus only appeared after a key press. Feedback was given upon an erroneous response (i.e. false denying recognition of the target items or falsely claiming recognition of the critical and irrelevant items), but the TOO SLOW message was not presented. Participants could proceed to the next phase when their target accuracy was at least 50%, otherwise the first practice phase was repeated until this requirement was met. In the second phase, the 1500 ms stimulus presentation was added, so that the next trial would automatically appear upon key press or after 1500 ms. Again, feedback was given upon an erroneous response, but the TOO SLOW message was never presented. Participants could only proceed to the next phase when their target accuracy was at least 50% and as an additional requirement, when their mean response latency was < 800 ms, otherwise this practice phase was repeated until their performance was satisfactory. The last practice phase was identical to the full test, including the WRONG and TOO SLOW feedback. Participants could proceed to the actual test only when their target accuracy was at least 50% and when their mean response latency was < 800 ms.

After completing the full CIT procedure consisting of 672 trials, participants were presented with a questionnaire designed to assess their attention to the tasks, involvement in the experiment, memory for the stimuli, and their motivation to avoid detection in the CIT on a 5-point Likert scale (ranging from 1 = not at all to 5 = very much so). Then, participants were told that the experiment was finished and they did not have to hide any information anymore. Finally, all participants completed the recall and recognition questionnaire assessing their memory of the robbery’s details, before being debriefed and compensated for participation with research credits.

## Results

Trials with an incorrect response (i.e. pressing NO for target items or pressing YES for either critical or irrelevant items)^2^ as well as trials with a RT < 150 ms or > 800 ms were excluded from analysis (see also Verschuere, Crombez, Degrootte, & Rosseel, 2010). On average, 617 trials (91.8%) per participant were included in the analyses (range 75.6–97.9%).

All analyses used an alpha level of 0.05. Effect sizes for the ANOVA are reported using Cohen’s *f*. For follow-up contrasts, Cohen’s *d* is used. As a rule of thumb, Cohen (1992) proposed 0.20, 0.50, and 0.80 as thresholds for “small,” “moderate,” and “large” effects, respectively, for *d* values and 0.10, 0.25, and 0.40 as thresholds for “small,” “moderate,” and “large” effects for *f* values. In addition, JZS Bayes factors were computed to further examine detection efficiency using JASP (JASP Team, 2018). Bayes factors are numerical values quantifying the odds ratio between the null and the alternative hypothesis given the data, with BF_01_ annotating how much more likely the data are under the null as compared to the alternative hypothesis and BF_10_ annotating how much more likely the data are under the alternative as compared to the null hypothesis. For one-tailed testing, Bayes factors are reported as either predicting the null (BF_0+_) or the alternative hypothesis (BF_+ 0_) in case of a positive effect, and BF_0−_ and BF_− 0_ for negative effects. A default JZS prior with scaling factor *r* = 0.707 was used for the alternative hypothesis (see Rouder, Speckman, Sun, Morey, & Iverson, 2009). Using Jeffreys’ (1961) criteria, a Bayes factor > 1, 3, 10, and 100 is taken as anecdotal, substantial, strong, and decisive evidence for the respective hypothesis.

### Confirmatory analyses: Concealed Information Test

The main analysis consisted of a 2 (Delay: immediate CIT versus one-week-delayed CIT, between-participants) by 2 (Abstractness level at encoding: items encoded on category level versus items encoded on exemplar level, within-participants) by 2 (Abstractness level in the CIT: items tested on category level versus items tested on exemplar level, within-participants) mixed ANOVA on the CIT-effect, calculated as the reaction time difference (RT_critical items_ – RT_irrelevant items_) in milliseconds.

The mixed ANOVA revealed a significant main effect of Abstractness level in the CIT, *F* (1, 134) = 6.24, *p* = 0.014, *f* = 0.21, that subsumed under the significant interaction between Abstractness level at encoding and Abstractness level in the CIT, *F* (1, 134) = 65.85, *p* < 0.001, *f* = 0.70 (i.e. larger RT difference when encoding and testing were on a congruent level). No significant main effect was found for Delay, *F* (1, 134) = 1.58, *p* = 0.210, *f* = 0.11.

There was no significant main effect of Abstractness level at encoding, *F* (1, 134) = 0.79, *p* = 0.376, *f* = 0.08. No significant interaction effect was found between Abstractness level at encoding and Delay, *F* (1, 134) = 0.72, *p* = 0.398, *f* = 0.07, or Abstractness level in the CIT and Delay, *F* (1, 134) = 0.10, *p* = 0.757, *f* = 0.03. Lastly, the three-way interaction did not reveal significant effects, *F* (1, 134) = 1.58e-5, *p* = 0.997, *f* = 0.00.

To narrow down the predicted Abstractness level at encoding by Abstractness level in the CIT interaction, planned contrasts were conducted across the immediate and delayed condition with Item Type as fixed factors (i.e. Category-Category, Category-Exemplar, Exemplar-Category, and Exemplar-Exemplar).^3^

A first planned contrast compared the RT difference between the Category-Exemplar Item Type with the three other Item Types to test the hypothesis that participants with categorical information only do not show recognition of the exemplar-level stimuli. The contrast revealed a significantly smaller CIT-effect in the Category-Exemplar Item Type compared to the three other Item Types in which knowledge existed, t (355.99) = 5.48, *p* < 0.001.^4^

A second planned contrast compared the RT difference of the Exemplar-Category Item Type with the two Item Types in which the abstractness level was the congruent for Encoding and Testing (i.e. Category-Category and Exemplar-Exemplar). The contrast revealed that the CIT-effect was significantly lower in the Exemplar-Category Item Type than for the other two Item Types, t (332.20) = 7.89, *p* < 0.001.^5^ Note that the Category-Exemplar Item Type was not included in these contrasts, since participants are not expected to have a larger response latency to the critical compared to irrelevant items.

Following the preregistration, an additional comparison was performed on the Category-Category versus Exemplar-Exemplar Item Types, to examine whether questions in the CIT are best asked on category or on the exemplar level when encoded in the congruent level. The two-tailed paired-samples *t*-test revealed that the CIT-effect for the Category-Category (*M* = 13.80, *SD* = 31.99) was smaller than that of the Exemplar-Exemplar Item Type (*M* = 20.82, *SD* = 33.55), *t* (135) = 2.19, *p* = 0.030, *d* = 0.19.^6^ However, an additional Bayesian two-tailed paired-samples *t*-test revealed that this difference was not sufficiently supported by the data, as the Bayes factor for the CIT-effect difference between Category-Category and the Exemplar-Exemplar Item Types was close to 1 (*BF* = 1.04 in favor of H_0_).^7^

Moreover, as per preregistration, a one-tailed Bayesian paired-samples *t*-test was performed on the critical-irrelevant contrast in each condition to investigate whether detection efficiency was above chance. Table 1 shows the mean scores for each cell of the design. For both the immediate and delayed CIT conditions, the results revealed the expected CIT-effect for the Item Types Category-Category and Exemplar-Exemplar, reflected by strong to decisive evidence that recognition of the critical item results in larger RTs to the critical compared to the irrelevant items. For the Item Type Exemplar-Category there was no CIT-effect, reflected by strong evidence for the null showing no generalization from the exemplar to the category level. For the two-tailed Category-Exemplar Item Type analysis, Bayesian statistics revealed anecdotal to substantial evidence for the null hypothesis. Indeed, as participants encoded the critical information at the categorical level, they were not expected to distinguish the correct exemplar from the irrelevant exemplars in the CIT.

**Table 1:** Mean reaction times (in ms) for the immediate and delayed condition per Item Type

Item Type		*M (SD)*	*d*_within (95% CI)_	BF	*M (SD)*	*d*_within (95% CI)_	BF
		Immediate CIT condition (*n* = 71)			Delayed CIT condition (*n* = 65)		
Category-Category	Critical	500 (56)	0.47 [0.24;0.71]	BF_+ 0_ = 407.31	493 (51)	0.37 [0.12;0.62]	BF_+ 0_ = 12.67
	Irrelevant	484 (43)			482 (49)		
Exemplar-Exemplar	Critical	503 (62)	0.55 [0.31;0.80]	BF_+ 0_ = 3368.63	497 (53)	0.69 [0.43;0.96]	BF_+ 0_ = 92,092.37
	Irrelevant	482 (49)			477 (50)		
Exemplar-Category	Critical	474 (50)	−0.23 [− 0.46;0.01]	BF_0+_ = 19.62	473 (61)	− 0.27 [− 0.51;-0.02]	BF_0+_ = 22.56
	Irrelevant	480 (43)			480 (53)		
Category-Exemplar	Critical	471 (49)	−0.06 [−0.28;0.16]	BF_01_ = 7.13	466 (53)	−0.23 [− 0.47;0.02]	BF_01_ = 1.12
	Irrelevant	472 (48)			471 (52)		

### Exploratory analyses

For all questions in the follow-up questionnaire, an independent-samples *t*-test was conducted to evaluate whether the ratings of participants in the immediate condition (*n* = 65) differed significantly from participants who completed the CIT after a one-week delay (*n* = 68). As predicted, participants in the immediate condition reported to have a better memory for details of the crime scenario than participants who were tested after a one-week delay. Participants in both conditions did not significantly differ in their focus, involvement, or effort to hide the critical information. Table 2 shows the mean scores for each cell of the design.

**Table 2:** Mean scores on the follow-up questionnaire (5-point Likert scale)

Question	*M (SD)*	*M (SD)*	*t*	*df*	*p*	*d*_between (95% CI)_
	Immediate condition (*n* = 71)	Delayed condition (*n* = 65)				
Focus	4.17 (0.74)	4.09 (0.72)	0.06	134	0.542	0.11 [−0.23;0.45]
Involvement	4.23 (0.90)	4.03 (0.79)	1.34	133.8	0.181	0.24 [−0.10;0.57]
Memory for robbery	4.37 (0.66)	4.07 (0.89)	2.17	134	0.032^a^	0.39 [0.04;0.72]
Memory for targets	4.17 (0.70)	4.25 (0.79)	0.61	134	0.546	−0.11 [−0.44;0.23]
Effort to conceal knowledge	4.65 (0.51)	4.62 (0.74)	0.30	134	0.765	0.05 [−0.28;0.38]

The letter a reflects a significant difference between the conditions

For all memory data, one-tailed independent-samples *t*-tests were conducted to evaluate whether the memory of participants in the immediate CIT condition (*n* = 71) was significantly higher than that for those tested after a one-week delay (*n* = 65). For the free recall, the number of correctly remembered details was counted (range 0–8). For the recognition check the items were scored as either incorrect (0) or correct (1) and summed (range 0–8). While participants in the immediate condition recalled more items than participants in the delayed condition, no differences emerged on recognition scores. Table 3 shows the mean scores for each cell of the design.

**Table 3:** Mean scores (SDs in parentheses) on the memory questionnaires (range 0-8)

Question	*M (SD)*	*M (SD)*	*t*	*df*	*p*	*d*_between (95% CI)_
	Immediate condition (*n* = 71)	Delayed condition (*n* = 65)				
Recall	7.70 (0.52)	7.03 (1.22)	4.11	84.62	< 0.001	0.72 [0.35;1.06]
Recognition	7.68 (0.73)	7.63 (0.74)	0.36	134.00	0.360	0.06 [−0.28;0.40]

## Discussion

The current study explored the most appropriate level of item abstractness for memory detection. While questions in the CIT can be phrased at the basic category-level, such as which type of vehicle was used to flee from the crime scene, in case of leakage of information to innocent suspects, it might be safer to ask for specific critical knowledge, such as the brand of the vehicle. Participant pairs planned a fictitious bank robbery, encoding the critical details on either categorical (e.g. car) or exemplar (e.g. Citroën) level. In the CIT, half of the encoded details were tested on the same abstractness level, while the other four items were replaced by its antagonistic level of abstractness, leading to a crossed design. Results indicate an interaction between encoding and testing, such that CIT validity is highest when there is a match (i.e. congruency) between how information is encoded and tested. Delay between planning the crime and the memory detection test did not influence these results.

### Item selection in the Concealed Information Test

While the idea of using response latency to index deception is almost a century old (see Luria, 1932; Marston, 1920), current measurement equipment made it possible to pick up on differences in milliseconds between truthfully and falsely denying knowledge of a crime detail. Since then, the potential of RTs to support the notion that lying is more demanding than truth-telling, and hence takes more time, has been validated (Suchotzki, Verschuere, Van Bockstaele, Ben-Shakhar, & Crombez, 2017). The present findings are consistent with the notion that lying is more demanding than truth-telling by showing enhanced RTs when denying recognition of previously learned critical details. Importantly, this pattern did not arise when responding to unknown exemplar items after encoding items merely at the category level. Both these patterns affirm the validity of memory detection using the RT measure to distinguish between knowledgeable and unknowledgeable individuals.

When general details from the crime are leaked to innocent suspects during interrogation or from the media, it could lead to a false indication of concealed information knowledge. Upon recognition of the crime details, the innocent suspect may show a distinct response pattern to the critical details relative to the well-matched alternative options. While such false-positive outcomes may be circumvented by probing for more specific knowledge, this could result in false negatives, as the true culprit might not recall such specific information from the crime. The current results indicate no decreased detection efficiency when encoding and testing specific items in the CIT as compared to using broader categories. While a statistically significant difference was found favoring the Exemplar-Exemplar Item Type over Categorical encoding and testing, Bayesian analysis was inconclusive. Additional research will be required to reach firm conclusions regarding the optimal balance between sensitivity and specificity, especially since extensive memory research has demonstrated that the categorical level is optimal for various cognitive functions, such as perception and most importantly, memory (Rosch, Mervis, Gray, Johnson, & Boyes-Braem, 1976). Yet, given the risk associated with information contamination, the current results suggest that the exemplar-level CIT could become a promising tool in the future when leakage is suspected.

#### Generalization

In the current study, no generalization occurred when details were encoded in its’ exemplar form (e.g. *Citroën*) and the CIT-question asked about the categorical getaway vehicle (e.g. *train*, *bike*, *taxi*, *car*, or *bus*). Earlier CIT research has demonstrated the sensibility of the CIT to detect partial information, such as synonyms of the critical detail. Moreover, Ben-Shakhar et al. (1996) showed a moderate degree of physiological generalization from items encoded at the exemplar level (e.g. *apple*) to its superordinate category level (e.g. *fruit*). In the current study, this effect was not replicated, neither in the immediate nor in the delayed condition.

We have speculated whether the absence of generalization could have been a result of the fast and cognitively demanding character of the RT-CIT in which all eight details were flashed on the screen in random order, either in its categorical or exemplar form. Consequently, participants might have not recognized the subordinate items as such, leading to unchanged behavior upon presentation of the critical items. However, an additional pilot study (*n* = 75) using the single-probe protocol of the RT-CIT (for the procedure, see Verschuere et al., 2015), in which all items belonging to a single question are presented sequentially before continuing to the next question, rather than presenting all items randomly intermixed, revealed similar results as the current study, including the lack of generalization.

Another possibility could lay in the fact that participants in both the immediate condition as well as after a one-week delay had a high recall and recognition ability of the eight critical items. Interestingly, memory research has indicated that especially over time, information is most likely retained at the basic category level (Pansky & Koriat, 2004). Recollection of specific details declines over time, while the gist of the event remains (Conway, Cohen, & Stanhope, 1991; Kintsch, Welsch, Schmalhofer, & Zimny, 1990). More specifically, a slower forgetting curve was found for basic level categorical information (e.g. *bird*), whereas individuals could not anymore distinguish between exemplar-level items (e.g. *sparrow* or *canary*; Dorfman & Mandler, 1994). It might be the case that since the memory for the original information was nearly perfect, the details were not yet converged to its categorical name, thereby explaining the lack of generalization from *Citroën* to *car*.

#### Delay

In contrast to the present results, several studies (Carmel et al., 2003; Gamer et al., 2010; Nahari & Ben-Shakhar, 2011) found an attenuated, yet significant CIT effect when the test was administered after a one- or two-week delay, which was more pronounced for peripheral items as opposed to details central to the crime. Although with a small sample, Hira, Sasaki, Matsuda, Furumitsu, and Furedy (2001, 2002) found that participants guilty of a mock-crime could be detected after a one-month and even a one-year delay. With the current results in mind, the categorical CIT might still be effective when administered sometime after the crime. Our findings showing no detrimental effect of delaying the CIT may be accounted for by our use of well-rehearsed items. However, as memory research has demonstrated optimal memory retention for the categorical level, further studies investigating the level of abstractness on which details are encoded, stored, and retrieved by culprits, should give more insight in the expected effects of a highly detailed CIT with regard to false-negative outcomes. While the current results indicate positive effects for the exemplar-level CIT after a one-week time delay in preventing false positives, further research should confirm its validity.

### Limitations and suggestions for future research

Questions can be raised about the success of the delay manipulation. While a statistically significant reduction in recall was revealed between participants who conducted the CIT immediately versus after a one-week delay, the size of the reduction was rather small, implying negligible practical significance. Furthermore, for both conditions, a clear ceiling effect can be observed in the recognition memory test, with no differences due to time delay. As the CIT is a test of recognition, this might explain the absence of an effect of delay in the current paradigm. Further research should elongate the time interval between the planning of the robbery and the memory detection test, as this is common practice in the field of deception detection.

Moreover, the encoding phase was very exhaustive and might have caused the ceiling effect in memory performance. This might be one possible reason why no generalization occurred from the exemplar to the category level. By studying the mock robbery on a more contextual level in future research, in contrast to the specific semantic setting of the current experiment, participants might also show increased responses to the word “*car*,” as opposed to “*Citroën*.” A successful time delay manipulation is expected to also influence the detailedness of the culprit’s memory, as memory performance declines over time. While the perpetrator might still remember that he fled the crime scene with the nearest parked *Citroën*, three months after the crime, this memory might have changed to the word “*car*,” unless the specific information was highly salient (e.g. when the *Citroën* was specifically chosen because of its discrete color or strong engine, it is very likely that this item is central to the crime and will be better stored in memory).

To further examine the external validity of the CIT, future studies should include a less strong encoding manipulation and/or a more realistic scenario, possibly presented in a visual or virtual-reality format. Moreover, as the current results based on RTs partially contradict previous results based on the electrodermal measure (Ben-Shakhar et al., 1996), it will be important to replicate the current experiment using psychophysiological measures. It should be noted that while the electrodermal measure reflects orienting response (see also klein Selle, Verschuere, Kindt, Meijer, & Ben-Shakhar, 2016, 2017), reaction time measures reflect different processes (e.g. cognitive load, inhibition, and response conflict). Consequently, the two types of measures can also be affected by different factors.

### Take home message

The current results indicate that what constitutes a good CIT question is very dependent on the encoding of the crime scene, thereby challenging examiners to formulate appropriate items to achieve an optimal balance between sensitivity and specificity.

## Additional files

Additional file 1: Item combinations in the crime scenario. (DOCX 21 kb)

Additional file 2: Exploratory analyses on error rates. (DOCX 24 kb)

## Footnotes

1 Participants were given the bonus in research credits when their individual CIT score (d_CIT_ = (M_RT(critical items)_ – M_RT(irrelevant items)_) / SD_RT(irrelevant items)_) was < 0.2 (see also Noordraven & Verschuere, 2013).

2 See online Additional file 2 for additional exploratory analyses on error rates.

3 For sake of readability and correctness, as there was no significant influence of a time delay on CIT performance, the reported contrasts were conducted across the immediate and delayed CIT condition. As this is a deviation from the preregistration, the planned contrasts separately for each condition can be found in footnotes.

4 For the immediate CIT condition, the planned contrast revealed that in the Category-Exemplar Item Type, in which no recognition was possible, there was a significantly smaller CIT-effect compared to the three other levels, *t* (218.49) = 3.79, *p* < 0.001. For the delayed condition, the planned contrast also revealed a significantly smaller CIT-effect in the Category-Exemplar Item Type compared to the three other Item Types, *t* (256) = 3.50, *p* = 0.001.

5 For the immediate CIT condition, the planned contrast revealed that the CIT-effect was significantly lower in the Exemplar-Category Item Type than for the other two Item Types, *t* (183.90) = 5.62, *p* < 0.001. For the delayed condition, the planned contrast also revealed that the CIT-effect was significantly lower in the Exemplar-Category Item Type than for the other two Item Types, *t* (256) = 5.60, *p* < 0.001.

6 The two-tailed paired-samples *t*-test revealed for the immediate condition that the CIT-effect for the Category-Category (*M* = 16.63, *SD* = 33.99) was not significantly different from the Exemplar-Exemplar Item Type (*M* = 21.18, *SD* = 37.56), *t* (70) = 0.98, *p* = 0.332, *d* = 0.12. For the delayed condition, the CIT-effect for the Category-Category (*M* = 10.71, *SD* = 29.61) was significantly smaller than that of the Exemplar-Exemplar Item Type (*M* = 20.42, *SD* = 28.83), *t* (64) = 2.22, *p* = 0.030, *d* = 0.27.

7 For the immediate CIT-condition, the Bayesian two-tailed paired-samples *t*-test revealed substantial evidence that there are no differences in the CIT-effect between the Category-Category and the Exemplar-Exemplar Item Types, *BF* = 4.85 in favor of H_0_. For the delayed condition, the Bayesian two-tailed paired-samples *t*-test revealed no conclusive result whether the CIT-effect for the Category-Category differed from the Exemplar-Exemplar Item Type, *BF* = 0.75 in favor of H_0_.
