# Supplementary material for: It’s a match!? Appropriate item selection in the Concealed Information Test
Source: Cogn Res Princ Implic. 2019 Apr 3;4:11. doi: 10.1186/s41235-019-0161-8 (PMC6447635; doi:10.1186/s41235-019-0161-8)
Supplement: Supplementary file 1 — Item combinations in the crime scenario. (DOCX 21 kb) [file 41235_2019_161_MOESM1_ESM.docx]

Additional file 1

| Item Type  Version 1 | Encoding Phase | Relevant item  in CIT | Irrelevant items  in CIT | Target  in CIT |
| --- | --- | --- | --- | --- |
| Exemplar - Exemplar | Ring | Ring | Earrings, Necklace, Bracelet, Brooch | Tiara |
|  | Attic | Attic | Kitchen, Bedroom, Bathroom, Basement | Garden |
| Category - Category | Sports | Sports | University, Bar, Work, Neighbours | Social Media |
|  | May | May | June, August, September, October | July |
| Exemplar - Category | SNS Bank | Bank | Museum, Gas station, Juweler, Cafetaria | Restaurant |
|  | Delft | South-Holland (province) | Friesland, Drenthe, Overijssel, Zeeland | Limburg |
| Category - Exemplar | Pointed Weapon | Butterfly knife | Pocket knife, Dagger, Stiletto knife, Hunting knife | Chef’s knife |
|  | Car | Citroën | Opel, Fiat, Ford, Peugeot | Volkswagen |

| Item Type  Version 2 | Encoding Phase | Relevant item  in CIT | Irrelevant items  in CIT | Target  in CIT |
| --- | --- | --- | --- | --- |
| Exemplar - Exemplar | SNS Bank | SNS Bank | Fortis Bank, ABN Amro, RABO Bank, ASN Bank | ING Bank |
|  | Delft | Delft | Rotterdam, The Hague, Leiden, Gouda | Scheveningen |
| Category - Category | Pointed Weapon | Pointed weapon | Fire-arm, Explosives, Taser, Blunt weapon | Chemical weapon |
|  | Car | Car | Bus, Scooter, Bike, Tram | Taxi |
| Exemplar - Category | Ring | Jewels | Cash, Negotiable instruments, coin collection, goldbars | Data/Information |
|  | Attic | At home | Friends house, at work, train station, warehouse | Park |
| Category - Exemplar | Sports | Volleybal | Soccer, hockey, basketbal, tennis | Gym |
|  | May | May 26S | May 8, May 12, May 17 May 22 | May 30 |

| Item Type  Version 3 | Learning Phase | Relevant | Irrelevants | Target |
| --- | --- | --- | --- | --- |
| Exemplar - Exemplar | Butterfly knife | Butterfly knife | Pocket knife, Dagger, Stiletto knife, Hunting knife | Chef’s knife |
|  | Citroen | Citroën | Opel, Fiat, Ford, Peugeot | Volkswagen |
| Category - Category | Bank | Bank | Museum, Gas station, Juweler, Cafetaria | Restaurant |
|  | South-Holland | South-Holland (province) | Friesland, Drenthe, Overijssel, Zeeland | Limburg |
| Exemplar - Category | Volleybal | Sports | University, Bar, Work, Neighbours | Social Media |
|  | May 26 | May | June, August, September, October | July |
| Category - Exemplar | Jewels | Ring | Earrings, Necklace, Bracelet, Brooch | Tiara |
|  | At home | Attic | Kitchen, Bedroom, Bathroom, Basement | Garden |

| Item Type  Version 4 | Learning Phase | Relevant | Irrelevants | Target |
| --- | --- | --- | --- | --- |
| Exemplar - Exemplar | Volleybal | Volleybal | Soccer, hockey, basketbal, tennis | Gym |
|  | May 26 | May 26 | May 8, May 12, May 17 May 22 | May 30 |
| Category - Category | Jewels | Jewels | Cash, Negotiable instruments, coin collection, goldbars | Data/Information |
|  | At home | At home | Friends house, at work, train station, warehouse | Park |
| Exemplar - Category | Butterfly knife | Pointed weapon | Fire-arm, Explosives, Taser, Blunt weapon | Chemical weapon |
|  | Citroen | Car | Bus, Scooter, Bike, Tram | Taxi |
| Category - Exemplar | Bank | SNS Bank | Fortis Bank, ABN Amro, RABO Bank, ASN Bank | ING Bank |
|  | South-Holland | Delft | Rotterdam, The Hague, Leiden, Gouda | Scheveningen |
