# Supplementary material for: It’s a match!? Appropriate item selection in the Concealed Information Test
Source: Cogn Res Princ Implic. 2019 Apr 3;4:11. doi: 10.1186/s41235-019-0161-8 (PMC6447635; doi:10.1186/s41235-019-0161-8)
Supplement: Supplementary file 2 — Exploratory analyses on error rates. (DOCX 24 kb) [file 41235_2019_161_MOESM2_ESM.docx]

**Additional file 2 – Error Analysis**

**Results**

Trials with an incorrect response (i.e., pressing NO for target items or pressing YES for either critical or irrelevant items) are excluded in the analysis.

All analyses used an alpha level of .05. Effect sizes for the ANOVA are reported using Cohen’s *f*. For follow-up contrasts Cohen’s *d* is used. As a rule of thumb, Cohen (1988) proposed 0.20, 0.50 and 0.80 as thresholds for “small”, “moderate” and “large” effects, respectively, for *d* values and 0.10, 0.25 and 0.40 as thresholds for “small”, “moderate” and “large” effects for *f* values.

**Concealed Information Test**

In the main analysis, a 2 (Delay: immediate CIT vs. 1-week-delayed CIT, between-participants) by 2 (Abstractness level at encoding: items encoded on category level vs. items encoded on exemplar level, within-participants) by 2 (Specificity of the CIT: items tested on category level vs. items tested on exemplar level, within-participants) mixed ANOVA was conducted on error rate differences (%Error_probes_ – %Errors_irrelevants_).

The mixed ANOVA revealed a significant main effect of Delay, *F*(1, 134) = 8.79, *p* = .004, *f* = 0.26, and a significant interaction effect between Abstractness level at encoding and Abstractness level in the CIT *F*(1, 134) = 97.34, *p* < .001, *f* = 0.85. These effects are collapsed under a statistically significant three-way interaction, *F*(1, 134) = 9.46, *p* = .003, *f* = 0.27.

There was no significant main effect of Abstractness level at encoding, *F*(1, 134) = 2.66, *p* = .105, *f* = 0.14, or Abstractness level in the CIT, *F*(1, 134) = 0.38, *p* = .539, *f* = 0.05. No significant interaction effects were found between Abstractness level at encoding and Delay, *F*(1, 134) = 0.10, *p* = .748, *f* = 0.03, or Abstractness level in the CIT and Delay, *F*(1, 134) = 0.05, *p* = .816, *f* = 0.00.

To narrow down the predicted Abstractness level at encoding by Abstractness level in the CIT interaction, planned contrasts were conducted for the immediate and delayed CIT condition separately with Item Type as fixed factors (i.e., Category-Category, Category-Exemplar, Exemplar-Category and Exemplar-Exemplar).

A first planned contrast compared the error rate difference between the Category-Exemplar Item Type with the three other Item Types to test the hypothesis that participants with categorical information do not show recognition of the exemplar leveled stimuli. For the immediate CIT condition, the planned contrast revealed that in the Category-Exemplar Item Type, in which no recognition was possible, there were significantly smaller error rate differences compared to the three other levels, *t*(249.38) = 8.04, *p* < .001. For the delayed condition, the planned contrast also revealed significantly smaller error rate differences in the Category-Exemplar Item Type compared to the three other Item Types, *t*(244.76) = 6.55, *p* < .001.

A second planned contrast compared the error rate difference of the Exemplar-Category Item Type with the two Item Types in which the Level of Specificity was the same for both Encoding and Testing (i.e., Category-Category and Exemplar-Exemplar). For the immediate CIT condition, the planned contrast revealed that the error rate difference was significantly lower in the Exemplar-Category Item Type than for the other two Item Types, *t*(194.00) = 6.81, *p* < .001. For the delayed condition, the planned contrast also revealed that the error rate difference was significantly lower in the Exemplar-Category Item Type than for the other two Item Types, *t*(163.92) = 3.55, *p* < .001. Note that the Category-Exemplar Item Type was not included in these contrasts, since participants are not expected to make more errors to the critical compared to irrelevant items.

An additional comparison was performed on the Category-Category versus Exemplar-Exemplar Item Types, to examine whether questions in the CIT are best asked on category or on the exemplar level when encoded in the respective level. A two-tailed paired-sample t-test revealed for the immediate condition that the error rate difference for the Category-Category (*M* = 8.36, *SD* = 9.15) was not significantly different from the Exemplar-Exemplar Item Type (*M* = 8.92, *SD* = 10.19), *t*(70) = 0.42, *p* = .675, *d* = 0.05. For the delayed condition, the error rate difference for the Category-Category (*M* = 4.24, *SD* = 7.39) was not significantly different from the Exemplar-Exemplar Item Type (*M* = 4.93, *SD* = 7.81), *t*(64) = 0.61, *p* = .543, *d* = 0.08.

For completion sake, a one-tailed Bayesian paired-samples *t*-test was performed on the critical-irrelevant contrast in each condition to investigate whether detection efficiency was above chance. Table 1 shows the mean scores for each cell of the design. For both the immediate and delayed CIT condition, the results revealed the expected CIT-effect for the Item Types Category-Category and Exemplar-Exemplar, reflected by decisive evidence that recognition of the critical item results in larger error rates to the critical compared to the irrelevant items. For the Item Type Exemplar-Category the results were inconclusive. For the two-tailed Category-Exemplar Item Type analysis, as expected since participants are in fact unknowledgeable of the exemplar level, there was no CIT-effect, with substantial evidence for the null hypothesis.

**Table 1. Mean error rates (in %) for the immediate and delayed condition per Item Type.**

| **Item Type** |  | ***M(SD)*** | ***d*_within_** | **BF** | ***M(SD)*** | ***d*_within_** | **BF** |  |
| --- | --- | --- | --- | --- | --- | --- | --- | --- |
|  |  | Immediate CIT condition (*n* = 71) | | | Delayed CIT condition (*n* = 65) | | | |
| Category-Category | Critical | 11.42 (10.15) | 0.91  [0.64;1.19] | BF_+0_ =  2.99e+8 | 7.03 (8.54) | 0.57  [0.31;0.83] | BF_+0_ =  2038.29 |  |
|  | Irrelevant | 3.06 (3.27) |  |  | 2.79 (3.56) |  |  |  |
| Exemplar-Exemplar | Critical | 12.22 (11.53) | 0.87  [0.61;1.14] | BF_+0_ =  7.72e+7 | 8.24 (8.28) | 0.63  [0.37;0.90] | BF_+0_ =  9986.49 |  |
|  | Irrelevant | 3.31 (3.59) |  |  | 3.31 (4.69) |  |  |  |
| Exemplar-Category | Critical | 3.92 (8.41) | 0.17  [-0.05;0.39] | BF_0+_ =  1.58 | 4.40 (5.85) | 0.20  [-0.04;0.44] | BF_0+_ =  1.14 |  |
|  | Irrelevant | 2.84 (3.50) |  |  | 3.26 (4.63) |  |  |  |
| Category-Exemplar | Critical | 2.52 (3.59) | 0.04  [-0.18;0.26] | BF_01_ =  7.25 | 2.09 (3.73) | -0.16  [-0.40;0.08] | BF_01_ =  3.32 |  |
|  | Irrelevant | 2.36 (2.18) |  |  | 2.47 (3.34) |  |  |  |
